# Mortality after transcatheter aortic valve replacement for aortic stenosis among patients with malignancy: a systematic review and meta-analysis

**DOI:** 10.1186/s12872-022-02651-4

**Published:** 2022-05-10

**Authors:** Muhammad Umer Siddiqui, Omar Yacob, Joey Junarta, Ahmed K. Pasha, Farouk Mookadam, Mamas A. Mamas, David L. Fischman

**Affiliations:** 1 Department of Internal Medicine, Thomas Jefferson University Hospitals, 833 Chestnut Street, Suite 701, Philadelphia, PA 19107 USA; 2 Cardiovascular Medicine, MercyOne North Iowa Heart Center, Mason City, IA USA; 3 Cardiovascular Medicine, UHS Wilson Medical Center, Johnson City, NY USA; 4 Cardiovascular Medicine, Mayo Clinic Health System, Phoenix, AZ USA; 5 Cardiovascular Research Group, Center for Prognosis Research, Keele University, Keele, UK; 6 Cardiovascular Medicine, Thomas Jefferson University Hospital, Philadelphia, PA USA

## Abstract

**Background:**

With advancements in cancer treatment, the life expectancy of oncology patients has improved. Thus, transcatheter aortic valve replacement (TAVR) may be considered as a feasible option for oncology patients with severe symptomatic aortic stenosis (AS). We aim to evaluate the difference in short- and long-term all-cause mortality in cancer and non-cancer patients treated with TAVR for severe AS.

**Methods:**

Medline, PubMed, and Cochrane Central Register of Controlled Trials were searched for relevant studies. Patients with cancer who underwent treatment with TAVR for severe AS were included and compared to an identical population without cancer. The primary endpoints were short- and long-term all-cause mortality.

**Results:**

Of 899 studies included, 8 met inclusion criteria. Cancer patients had significantly higher long-term all-cause mortality after TAVR when compared to patients without cancer (risk ratio [RR] 1.43; 95% confidence interval (CI) 1.26–1.62; *P* < 0.01). Four studies evaluated short-term mortality after TAVR and demonstrated no difference in it in patients with and without cancer (RR 0.72; 95% CI 0.47–1.08; *P* = 0.11).

**Conclusion:**

Patients with cancer and severe AS have higher long-term all-cause mortality after TAVR. However, we found no difference in short-term all-cause mortality when comparing patients with and without cancer. The decision to perform TAVR in cancer patients should be individualized based on life expectancy and existing co-morbidities.

**Supplementary Information:**

The online version contains supplementary material available at 10.1186/s12872-022-02651-4.

## Introduction

Due to the lower risk of complications, transcatheter aortic valve replacement (TAVR) has become the treatment of choice over surgical aortic valve replacement (SAVR) for frail patients with symptomatic aortic stenosis (AS) [1]. The incidence of AS and cancer increases with age. Twenty six percent of patients with AS have a history of cancer or have active cancer [2, 3]. The increased incidence of both AS and cancer with age is expected due to shared risk factors related to cancer, cardiovascular disease, and the pathophysiology behind degenerative AS [4, 5]. It is known that radiotherapy, particularly mediastinal radiation for lymphoma, is associated with progressive aortic disease [6]. Concomitant chemotherapy, such as with anthracyclines, can further increase the incidence of AS [7]. Studies have shown that such therapy causes AS by inducing valvular degeneration [8]. Since cancer patients are at greater risk of developing AS, investigating TAVR outcomes in this population becomes crucial.

TAVR has been proven to improve the hemodynamics and functional status of patients with severe symptomatic AS [9–11]. Its widespread use has grown significantly. At the same time, the life expectancy of cancer patients has improved with advances in cancer therapy. As life expectancy increases in cancer patients, the presence of severe symptomatic AS may impact prognosis to a greater extent than that of many cancers. There have been limited studies that assess the mortality of cancer patients with AS after TAVR. Two previous meta-analyses on this topic were limited in scope and did not include all the available evidence in their pooled outcomes. Thus, we aim to comprehensively investigate the utility of TAVR in cancer patients with severe AS.

## Methods

### Data sources and search strategy

This systematic review and meta-analysis was reported according to Preferred Reporting Items for Systematic Review and Meta-Analyses (PRISMA) guidelines [12]. Medline, PubMed, and Cochrane Central Register of Controlled Trials were searched from database inception through December 2020 using the following combination of keywords: transcatheter aortic valve replacement OR transcatheter aortic valve implantation OR heart valve prosthesis AND mortality OR short-term mortality OR long-term mortality AND malignancy OR cancer OR neoplasms. No time restriction was placed on the search. However, language was restricted to English. To identify grey literature, online libraries including www.clinicaltrialresults.org, www.clinicaltrials.gov, and presentations from major cardiovascular proceedings were also searched. All citations retrieved from the search were transferred to EndNote X7.5 Reference Manager (Thompson ISI ResearchSoft, Philadelphia, Pennsylvania) and duplicates were removed.

### Study selection

All citations were screened by two independent reviewers (MUS and OY) on the basis of eligibility criteria. Inclusion criteria in the included studies comprised of adults with a diagnosis of cancer identified to have severe AS and underwent treatment with TAVR. Patients in whom TAVR was contraindicated or did not have TAVR performed and patients who had SAVR for treatment of severe AS were excluded. Studies that did not compare TAVR outcomes in patients without a history of active cancer were excluded. The primary endpoint in the included studies comprised of short- and long-term all-cause mortality. Short-term mortality was defined as death within 30 days after TAVR. Long-term mortality was defined as death 30 days after TAVR. Secondary analyses were performed to identify the risk of cardiac mortality, myocardial infarction (MI), stroke, acute kidney injury (AKI), and major bleeding among patients with and without cancer after TAVR.

### Data extraction

Two independent reviewers (MUS and OY) extracted the data on year of publication, study design, inclusion criteria, primary endpoints, type of cancer, and follow-up time using a standardized data extraction form.

### Statistical analysis

Outcomes from each study were pooled and compared using a random effects model to account for potential between study variances. Treatment effect was reported as risk ratio (RR) and was supplemented by 95% confidence intervals (CI). The I^2^-statistic was quantified to measure heterogeneity with values > 25%, 50%, and 75% consistent with low, moderate, and high degrees of heterogeneity, respectively [13]. Review Manager Software v5.41 was used for the analysis. A funnel plot was used to assess for publication bias. *P*-values less than 0.05 were considered statistically significant. Certainty in the evidence (i.e., confidence in the final estimates), was assessed using the GRADE approach (Grades of Recommendation, Assessment, Development, and Evaluation) based on the risk of bias, imprecision, indirectness, inconsistency, and publication bias.

### Quality assessment of the included studies

Risk of bias was assessed using the Modified Newcastle–Ottawa scale for observational studies, which assesses 3 domains: patient selection, comparability, and outcome assessment (Additional file 1: Table S1) [14]. The methodological quality of a study was graded as high or low based on whether the study had adequate adjustment for confounders, which we judged to be the most critical domain affecting the main outcomes of interest [15].

## Results

### Baseline demographics

**Fig. 1:**
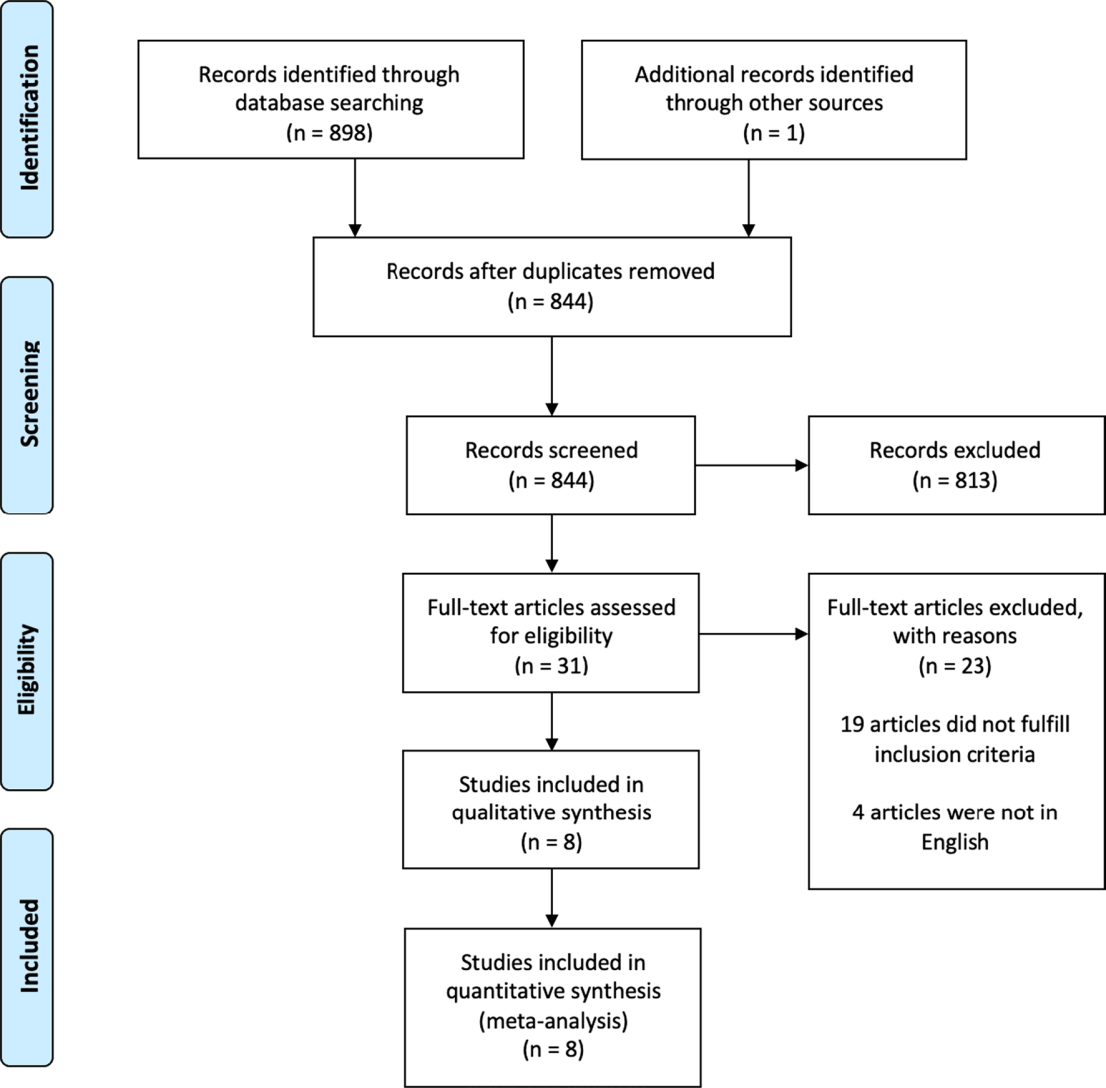
The preferred reporting items for systematic reviews and meta-analyses (PRISMA) flow diagram of the included studies

After exclusion of duplicate and irrelevant items, the initial search resulted in 899 articles. Eight studies with a total of 12,165 patients met inclusion criteria for quantitative analysis (Fig. 1) [16–23]. All the studies included were observational. The baseline characteristics of the included studies are shown in Table 1. Mean age ranged between 79 and 83 in the cancer group and 81–85 in the non-cancer group (Additional file 1: Table S2). The mean follow-up time period was 2.4 years. Both solid and hematologic malignancies were included in the studies. Transfemoral access was the most common approach utilized for TAVR. Bleiziffer et al. and Tabata et al. did not report the approach utilized for TAVR, while Mangner et al. included only patients who underwent TAVR with a transfemoral approach. The remaining studies included TAVR procedures utilizing transapical, transaxillary, and transiliac approaches [16–18, 20]. The prospective study conducted by Watanabe et al. used only balloon-expandable valves, whereas the study performed by Landes et al. and Bleiziffer et al. utilized self-expandable valves. Biancari et al. did not report the type of valve utilized for TAVR. The study conducted by Watanabe et al. and Landes et al. included patients with only active cancer, while Berkovitch et al., Bleiziffer et al. and Nuis et al. included patients only with past malignancy. In contrast, Mangner et al. and Biancari et al. included patients with both active and past cancer. Outcome data from the included studies are summarized in Additional file 1: Table S3.

**Table 1:** Baseline characteristics of the included studies

Author, year	Study design	Inclusion criteria	Primary endpoint	Type of cancer	Follow-up duration	TAVR approach	Valve type	Exclusion criteria
Mangner, 2018	Prospective non-randomized trial	Patients with active cancer, history of cancer and without cancer who underwent TAVR	All-cause mortality	Solid (predominantly breast and prostate cancer) and hematologic	3 years	Transfemoral	Self-expandable and balloon-expandable	NR
Watanabe, 2016	Prospective non-randomized trial	Patients who underwent TAVR for symptomatic severe AS	Post-procedural outcomes	Solid (lung, prostate, breast, colon, liver, head and neck, gastric, pancreatic, renal, bladder, thyroid, brain) and hematologic	2 years	Transfemoral, transiliac, transapical	Edwards Sapien	Bicuspid aortic valve, failed SAVR, severe AR and dialysis dependence
Landes, 2019	Prospective non-randomized trial	Patients with active cancer who underwent TAVR	All-cause mortality	Solid (gastrointestinal, prostate, breast, lung, renal, bladder) and hematologic	1 year	Transfemoral	Self-expandable	NR
Berkovitch, 2018	Prospective non-randomized trial	Patients who underwent TAVR for symptomatic severe AS	All-cause mortality	Solid and hematologic	2.3 years	Transfemoral, transapical, transaxillary	Medtronic CoreValve and Edwards Sapien	NR
Biancari, 2020	Retrospective study	Patients who underwent TAVR for symptomatic severe AS	All-cause mortality, cardiovascular mortality	Solid and hematologic	Mean 2.1 years	Transapical	NR	NR
Bleiziffer, 2017	Prospective adjusted non-randomized trial	Patients who underwent TAVR for symptomatic severe AS	MACE	NR	Mean 2.4 years	NR	Medtronic CoreValve	Enrollment in another trial or inability to give informed consent
Nuis, 2013	Observational adjusted study	Patients who underwent TAVR for symptomatic severe AS	Mortality	NR	1 year	Transfemoral, transapical, transsubclavian, transaortic	Medtronic CoreValve, Edwards Sapien, Direct Flow Valve	Patients with missing baseline hemoglobin
Tabata, 2020	Observational cohort study	Patients who underwent TAVR for symptomatic severe AS	All-cause mortality	Solid (predominantly breast, prostate, and colon cancer) and hematologic	5 years	NR	Self-expandable and balloon-expandable	NR

TAVR, transcatheter aortic valve replacement; NR, not reported; AS, aortic stenosis; SAVR, surgical aortic valve replacement; AR, aortic regurgitation; MACE, major adverse cardiovascular events

### Short-term all-cause mortality

**Fig. 2:**
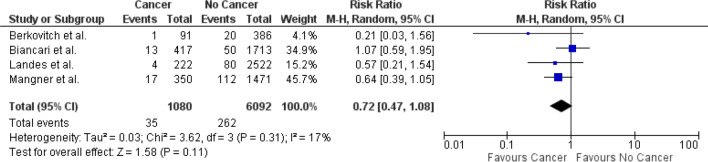
Forest plot for short-term mortality comparing patients with and without cancer who underwent TAVR. *Legend*: The pooled risk ratio with 95% confidence intervals were calculated using a random effects model. Weight refers to the contribution of each study to the pooled estimate. Squares and horizontal lines denote the point estimate and 95% confidence interval for each study’s risk ratio. The diamond signifies the pooled risk ratio; the diamond center denotes the point estimate and the width denotes the 95% confidence interval

Four studies reported short-term all-cause mortality associated with TAVR in patients with cancer [17–19, 21]. The data for meta-analysis was pooled from these studies. The risk of short-term mortality was not significantly different among TAVR patients with and without cancer (RR 0.72; 95% CI 0.47–1.08; Fig. 2). Very little variation was noted between the trials as indicated by low I^2^ value of 17%.

### Long-term all-cause mortality

**Fig. 3:**
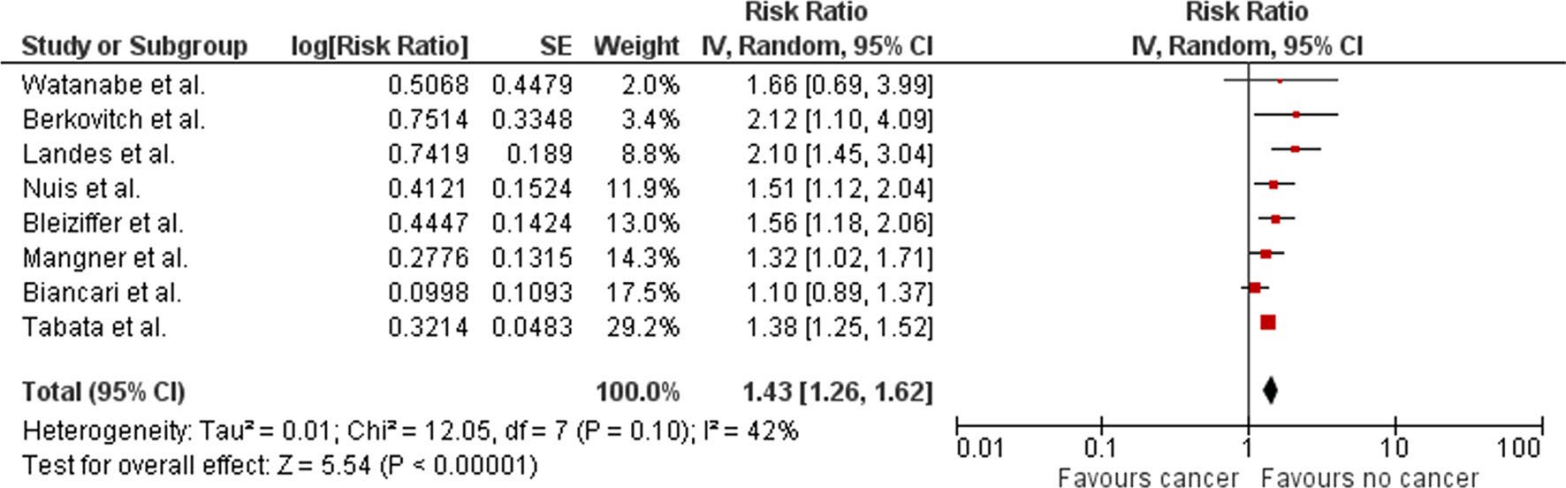
Forest plot for long-term mortality comparing patients with and without cancer who underwent TAVR. *Legend*: The pooled risk ratio with 95% confidence intervals were calculated using a random effects model. Weight refers to the contribution of each study to the pooled estimate. Squares and horizontal lines denote the point estimate and 95% confidence interval for each study’s risk ratio. The diamond signifies the pooled risk ratio; the diamond center denotes the point estimate and the width denotes the 95% confidence interval

Eight studies reported long-term mortality associated with TAVR in patients with cancer [17–23]. Pooled results of these studies identified significantly higher risk of long-term mortality among TAVR patients with cancer when compared to patients without cancer (RR 1.43; 95% CI 1.26–1.62; Fig. 3). Low level of variation was noted between the trials in the primary analysis as indicated by I^2^ values of 42%.

### Sensitivity and subgroup analyses

Sensitivity analyses of long term all-cause mortality was performed to identify if the trend was similar to the overall result. For this purpose, the included studies were organized into unadjusted and adjusted studies. The sensitivity analysis identified that the difference in long term all-cause mortality remained statistically significant in both unadjusted (RR 1.68, 95% CI 1.26–2.25) and adjusted (RR 1.35, 95% CI 1.18–1.55) subgroups similar to the overall result (Additional file 1: Figure S1). For subgroup analysis, the study population was organized into no cancer (NC) (control group), active cancer (AC), and past cancer (PC) groups. Similar to the pooled result, there was no difference in short-term mortality among patients with AC and NC. However, there was significantly higher risk of short-term mortality in the NC group when compared to the PC group (Additional file 1: Figure S2). This contrasting result was likely due to the small sample size and increased confounding in the unadjusted studies. Similar to the pooled result, there was significantly higher risk of long-term mortality in patient with AC and PC when compared to NC (Additional file 1: Figure S3). Further subgroup analysis was performed for long term mortality by classifying the studies into those with follow-up of 2 years or less and follow-up of greater than 2 years. Both the groups showed increased risk of long term mortality among cancer patients (RR 1.72; 95% CI 1.37–2.15 and RR 1.35; 95% CI 1.19–1.53) (Additional file 1: Figure S4).

### Leave one out analysis

After removing the study performed by Landes et al. which included subjects with only active cancer, the result for long term mortality remained similar to the overall pooled result (RR 1.37; 95% CI 1.25–1.51). However, the heterogeneity decreased (I^2^ = 15%) (Additional file 1: Figure S5).

### Secondary endpoints

The results from included studies were pooled where data was available to identify the risk of secondary endpoints. There was no difference in long term cardiac mortality (RR 0.87, 95% CI 0.71–1.06), MI (RR 1.20, 95% CI 0.37–2.89), stroke (RR 0.89, 95% CI 0.59–1.35), AKI (RR 0.92, 95% CI 0.66–1.30), or major bleeding (RR 1.26, 95% CI 0.70–2.28) between patients with and without cancer who underwent TAVR (Additional file 1: Figures S6 and S7).

### Certainty in the estimates

The included studies were observational with variable methodological quality and thus are at increased risk of selection and confounding bias. The estimates were precise for short term mortality, long term mortality, and all the secondary endpoints except for MI which had less than 100 events. There was no indirectness or evidence of publication bias. Heterogeneity was noted among the included studies. The quantified I^2^ value for each individual primary outcomes investigated in this meta-analysis are as follows: short term mortality 17% (minimal) and long term mortality 42% (low). The I^2^ value for secondary outcomes ranged from 0 to 83%. Overall, the certainty in the estimates in all the outcomes was judged to be moderate. Additional file 1: Figure S8 demonstrates a funnel plot to assess for publication bias in the studies reporting long-term mortality.

## Discussion

We investigated the short- and long-term all-cause mortality in patients undergoing TAVR for AS with underlying malignancy compared to those without. There was no difference in short-term mortality among patients with cancer compared to those without who underwent TAVR. However, patients with malignancy had increased long-term all-cause mortality after TAVR. Subgroup analyses demonstrated that the higher risk of all-cause long-term mortality was apparent in those with active and past cancer. No significant difference was noted in the secondary endpoints between groups, including long-term cardiac mortality.

Our analysis differs from two previous meta-analyses published on this topic. The meta-analysis performed by Murphy et al. included studies that exclusively enrolled patients who received thoracic irradiation for cancer treatment [24]. These patients are expected to have higher cardiovascular complications, including constrictive pericarditis, coronary artery disease, conduction abnormalities, and valvular abnormalities when compared to chemotherapy related cardiac dysfunction [25]. Murphy et al. also did not include the studies performed by Landes, Biancari, and Watanabe et al. in their pooled analysis. In contrast to our study, Murphy et al. did not find a difference in long-term all-cause mortality in patients with and without cancer who underwent TAVR. Bendary et al. also performed a meta-analysis looking at mortality outcomes in cancer patients with TAVR [26]. However, the pooled analysis only included three studies and subgroup analysis was not performed to identify differences in outcomes comparing patients with active versus past cancer. The pooled results for short- and long term all-cause mortality was similar to our study.

The introduction of TAVR has allowed physicians to treat many patients with AS in whom aortic valve replacement (AVR) was initially thought to be contraindicated. Namely due to the risks and potential complications associated with open surgery. Severe symptomatic AS has a prognosis that is worse than many cancers with respect to both morbidity and mortality. The prognosis and expected length of survival is further worsened when patients with severe AS also have comorbid cancer. This raises the question whether these patients who have malignancy along with severe AS should be offered AVR.

The treatment of cancer, which includes oncologic surgery, chemotherapy, or radiation therapy, might lead to worsening of aortic valve disease, either because of effects on the valve or on myocardial function. In turn, this may result in withholding effective cancer therapy in patients suffering from severe AS. The European Society of Cardiology recommends afterload reduction with medical therapy in patients with left ventricle dysfunction or heart failure induced by anthracycline or antineoplastic therapy [27]. The most effective afterload reduction strategy in patients with AS is treatment of the stenotic valve, which can be through TAVR, SAVR, or balloon valvuloplasty. It has been observed that balloon valvuloplasty fails to improve survival in patients with AS, rather, it is associated with increased complications and higher restenosis rates [28, 29]. SAVR is usually decided on a case by case basis, but in patients with malignancy, concerns regarding important complications exist. Cancer patients undergoing SAVR may be at increased risk of infection due to immunosuppression, while cachexia may impact recovery and mediastinal fusion. Cancer patients are often anemic, have low platelet counts, and have clotting abnormalities [30]. This places them at higher risk of bleeding complications, particularly those placed on cardio-pulmonary bypass [31, 32]. Thus, the invasive nature of SAVR renders it less desirable in this patient population. TAVR might be the optimal strategy for the treatment of select oncology patients, as it minimizes the concerns associated with surgery, including with regards to its invasiveness, increased risk of bleeding, infections, and the suspension of oncological treatment after surgery during recovery [30–34].

Our study agrees with the findings from Mangner et al., Nuis et al., and Bleiziffer et al., who reported that malignancy was associated with increased odds of long-term mortality post TAVR. This is in contrast to the prospective studies by Watanabe et al. and Biancari et al., where they showed no difference in long-term mortality in patients with or without malignancy post-TAVR. We believe that this difference is likely due to the variability in cancer type and stage, duration of treatment, and ejection fraction (EF) in the population studied. It is important to recognize that long-term mortality post-TAVR is unlikely to be related to the TAVR procedure itself, but more likely to be driven by underlying pre-existing comorbidities. Participants included in the trial conducted by Watanabe et al. had a higher mean EF in both cancer and non-cancer groups compared to the study conducted by Mangner et al. Berkovitch et al. reported that patients with malignancy who underwent cancer related treatment < 1 year ago had a higher long-term mortality after TAVR [18]. Thus, this suggests that cancer activity significantly impacted patient survival. Further studies would be useful to clarify the role of cancer type and cancer stage on morbidity and mortality post-TAVR. Indeed, the 2021 European Society of Cardiology and the European Association for Cardiothoracic Surgery guidelines for the management of valvular heart disease recommends early intervention in those with symptomatic severe AS, except for those in whom intervention is unlikely to improve quality of life or survival or for those with concomitant conditions associated with survival < 1 year (e.g. malignancy).

We found no difference in periprocedural complications in patients with and without cancer after TAVR. Despite this, it is important to be conscious that performing TAVR in patients with cancer is still high-risk. These patients are at greater risk of cardiopulmonary dysfunction from prior chemoradiotherapy. Additionally, they are at increased risk of significant aortic valve and annular calcification. This makes treatment with self-expanding prostheses challenging due to under-expansion, which places patients at higher odds of paravalvular regurgitation [35, 36].

This meta-analysis has limitations primarily due to limitations in the studies that were included. The studies are non-randomized, introducing the possibility of selection and sample biases. There was a difference in baseline characteristics, including baseline cardiac function, malignancy type, TAVR approach, valve type and follow-up duration, which introduces heterogeneity. This limitation was reduced by performing subgroup and leave one out analyses. Meta-regression could not be performed due to the number of studies being less than 10. As the studies were not blinded, a moderate risk of performance bias was observed among the included studies. Finally, we restricted this study to include articles from PubMed and Cochrane databases. Hence, it is possible that there are other studies matching our inclusion criteria that are not included in our meta-analysis.

## Conclusion

This study offers insight into the mortality among cancer patients who undergo TAVR. Our meta-analysis identified higher risk of long-term all-cause mortality among patients with active and past cancer who undergo TAVR. The increased mortality is likely multifactorial and could be related to cancer stage, cancer type, chemotherapy utilized, and pre-existing co-morbidities. There was no difference in short-term mortality, cardiac mortality, or periprocedural complications between cancer and non-cancer patients who undergo TAVR. A multidisciplinary approach, including with oncologists and cardiac surgeons, is required to create a comprehensive plan for cancer patients being considered for TAVR. The decision to undergo TAVR in this population should always be individualized after contemplating the risks associated with the procedure as well as complications that could arise due to cancer.

## Supplementary Information

**Additional file 1.** Supplementary Material.

## Data Availability

Data is safely kept in a password protected security system at Thomas Jefferson University Hospital. All data generated or analysed during this study are included in this published article [and its additional files]. Code availability: Not applicable.

## References

[1] Gilard M, Eltchaninoff H, Iung B, Donzeau-Gouge P, Chevreul K, Fajadet J (2012). Registry of transcatheter aortic-valve implantation in high-risk patients. N Engl J Med.

[2] Mistiaen WP, Van Cauwelaert P, Muylaert P, Wuyts F, Harrisson F, Bortier H (2004). Effect of prior malignancy on survival after cardiac surgery. Ann Thorac Surg.

[3] Yusuf SW, Sarfaraz A, Durand JB, Swafford J, Daher IN (2011). Management and outcomes of severe aortic stenosis in cancer patients. Am Heart J.

[4] Johnson CB, Davis MK, Law A, Sulpher J (2016). Shared risk factors for cardiovascular disease and cancer: implications for preventive health and clinical care in oncology patients. Can J Cardiol.

[5] Mookadam F, Jalal U, Wilansky S (2010). Aortic valve disease: preventable or inevitable?. Future Cardiol.

[6] Aleman BM, van den Belt-Dusebout AW, De Bruin ML, van’t Veer MB, Baaijens MH, de Boer JP (2007). Late cardiotoxicity after treatment for Hodgkin lymphoma. Blood.

[7] Wethal T, Lund MB, Edvardsen T, Fosså SD, Pripp AH, Holte H (2009). Valvular dysfunction and left ventricular changes in Hodgkin's lymphoma survivors. A longitudinal study. Br J Cancer.

[8] Murbraech K, Wethal T, Smeland KB, Holte H, Loge JH, Holte E (2016). Valvular dysfunction in lymphoma survivors treated with autologous stem cell transplantation: a national cross-sectional study. JACC Cardiovasc Imaging.

[9] Carabello BA (2008). Aortic stenosis: a fatal disease with but a single cure. JACC Cardiovasc Interv.

[10] Sharma S, Mehra A, Rahimtoola SH (2008). Valvular heart disease: a century of progress. Am J Med.

[11] Bach DS, Cimino N, Deeb GM (2007). Unoperated patients with severe aortic stenosis. J Am Coll Cardiol.

[12] Moher D, Liberati A, Tetzlaff J, Altman DG (2009). Preferred reporting items for systematic reviews and meta-analyses: the PRISMA statement. Ann Intern Med.

[13] Turner RM, Davey J, Clarke MJ, Thompson SG, Higgins JP (2012). Predicting the extent of heterogeneity in meta-analysis, using empirical data from the Cochrane Database of Systematic Reviews. Int J Epidemiol.

[14] Stang A (2010). Critical evaluation of the Newcastle–Ottawa scale for the assessment of the quality of nonrandomized studies in meta-analyses. Eur J Epidemiol.

[15] Viswanathan M, Patnode CD, Berkman ND, Bass EB, Chang S, Hartling L (2018). Recommendations for assessing the risk of bias in systematic reviews of health-care interventions. J Clin Epidemiol.

[16] Watanabe Y, Kozuma K, Hioki H, Kawashima H, Nara Y, Kataoka A (2016). Comparison of results of transcatheter aortic valve implantation in patients with versus without active cancer. Am J Cardiol.

[17] Landes U, Iakobishvili Z, Vronsky D, Zusman O, Barsheshet A, Jaffe R (2019). Transcatheter aortic valve replacement in oncology patients with severe aortic stenosis. JACC Cardiovasc Interv.

[18] Berkovitch A, Guetta V, Barbash IM, Fink N, Regev E, Maor E (2018). Favorable short-term and long-term outcomes among patients with prior history of malignancy undergoing transcatheter aortic valve implantation. J Invasive Cardiol.

[19] Mangner N, Woitek FJ, Haussig S, Holzhey D, Stachel G, Schlotter F (2018). Impact of active cancer disease on the outcome of patients undergoing transcatheter aortic valve replacement. J Interv Cardiol.

[20] Nuis RJ, Sinning JM, Rodés-Cabau J, Gotzmann M, van Garsse L, Kefer J (2013). Prevalence, factors associated with, and prognostic effects of preoperative anemia on short- and long-term mortality in patients undergoing transcatheter aortic valve implantation. Circ Cardiovasc Interv.

[21] Biancari F, Dahlbacka S, Juvonen T, Virtanen MPO, Maaranen P, Jaakkola J (2020). Favorable outcome of cancer patients undergoing transcatheter aortic valve replacement. Int J Cardiol.

[22] Bleiziffer S, Bosmans J, Brecker S, Gerckens U, Wenaweser P, Tamburino C (2017). Insights on mid-term TAVR performance: 3-year clinical and echocardiographic results from the CoreValve ADVANCE study. Clin Res Cardiol.

[23] Tabata N, Al-Kassou B, Sugiura A, Kandt J, Shamekhi J, Stundl A (2020). Prognostic impact of cancer history in patients undergoing transcatheter aortic valve implantation. Clin Res Cardiol.

[24] Murphy AC, Koshy AN, Cameron W, Horrigan M, Kearney L, Yeo B (2021). Transcatheter aortic valve replacement in patients with a history of cancer: periprocedural and long-term outcomes. Catheter Cardiovasc Interv.

[25] Jaworski C, Mariani JA, Wheeler G, Kaye DM (2013). Cardiac complications of thoracic irradiation. J Am Coll Cardiol.

[26] Bendary A, Ramzy A, Bendary M, Salem M (2020). Transcatheter aortic valve replacement in patients with severe aortic stenosis and active cancer: a systematic review and meta-analysis. Open Heart.

[27] Zamorano JL, Lancellotti P, Rodriguez Munoz D, Aboyans V, Asteggiano R, Galderisi M (2016). 2016 ESC position paper on cancer treatments and cardiovascular toxicity developed under the auspices of the ESC Committee for Practice Guidelines: The Task Force for cancer treatments and cardiovascular toxicity of the European Society of Cardiology (ESC). Eur Heart J.

[28] Cubeddu RJ, Jneid H, Don CW, Witzke CF, Cruz-Gonzalez I, Gupta R (2009). Retrograde versus antegrade percutaneous aortic balloon valvuloplasty: immediate, short- and long-term outcome at 2 years. Catheter Cardiovasc Interv.

[29] Lieberman EB, Bashore TM, Hermiller JB, Wilson JS, Pieper KS, Keeler GP (1995). Balloon aortic valvuloplasty in adults: failure of procedure to improve long-term survival. J Am Coll Cardiol.

[30] Samuels LE, Kaufman MS, Morris RJ, Styler M, Brockman SK (1999). Open heart surgery in patients with chronic lymphocytic leukemia. Leuk Res.

[31] Ascione R, Williams S, Lloyd CT, Sundaramoorthi T, Pitsis AA, Angelini GD (2001). Reduced postoperative blood loss and transfusion requirement after beating-heart coronary operations: a prospective randomized study. J Thorac Cardiovasc Surg.

[32] Despotis GJ, Filos KS, Zoys TN, Hogue CW, Spitznagel E, Lappas DG (1996). Factors associated with excessive postoperative blood loss and hemostatic transfusion requirements: a multivariate analysis in cardiac surgical patients. Anesth Analg.

[33] Scott BH, Seifert FC, Glass PS, Grimson R (2003). Blood use in patients undergoing coronary artery bypass surgery: impact of cardiopulmonary bypass pump, hematocrit, gender, age, and body weight. Anesth Analg.

[34] Chan J, Rosenfeldt F, Chaudhuri K, Marasco S (2012). Cardiac surgery in patients with a history of malignancy: increased complication rate but similar mortality. Heart Lung Circ.

[35] Wu W, Masri A, Popovic ZB, Smedira NG, Lytle BW, Marwick TH (2013). Long-term survival of patients with radiation heart disease undergoing cardiac surgery: a cohort study. Circulation.

[36] Almeida JG, Ferreira SM, Fonseca P, Dias T, Guerreiro C, Barbosa A (2018). Comparison of self-expanding and balloon-expandable transcatheter aortic valves morphology and association with paravalvular regurgitation: Evaluation using multidetector computed tomography. Catheter Cardiovasc Interv.

