# Supplementary material for: Mortality after transcatheter aortic valve replacement for aortic stenosis among patients with malignancy: a systematic review and meta-analysis
Source: BMC Cardiovasc Disord. 2022 May 10;22:210. doi: 10.1186/s12872-022-02651-4 (PMC9088110; doi:10.1186/s12872-022-02651-4)
Supplement: Supplementary file 1 — Additional file 1. Supplementary Material. [file 12872_2022_2651_MOESM1_ESM.docx]

**Supplemental Figures**

**Supplemental Figure 1.** Forest plot for long-term mortality comparing patients with and without cancer who underwent TAVR in adjusted and unadjusted subgroups.


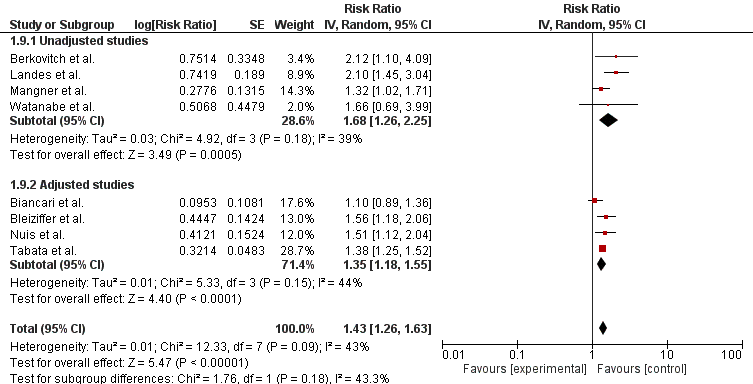


Legend: The pooled risk ratio with 95% confidence intervals were calculated using a random effects model. Weight refers to the contribution of each study to the pooled estimate. Squares and horizontal lines denote the point estimate and 95% confidence interval for each study’s risk ratio. The diamond signifies the pooled risk ratio; the diamond center denotes the point estimate and the width denotes the 95% confidence interval.

**Supplemental Figure 2.** Forest plot for short-term mortality comparing patients with active cancer versus past cancer who underwent TAVR.


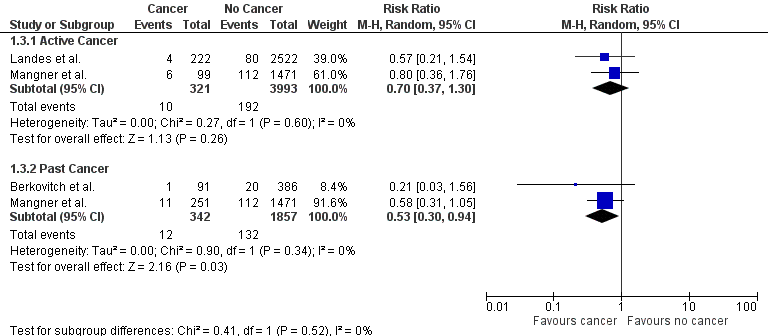


Legend: The pooled risk ratio with 95% confidence intervals were calculated using a random effects model. Weight refers to the contribution of each study to the pooled estimate. Squares and horizontal lines denote the point estimate and 95% confidence interval for each study’s risk ratio. The diamond signifies the pooled risk ratio; the diamond center denotes the point estimate and the width denotes the 95% confidence interval.

**Supplemental Figure 3.** Forest plot for long-term mortality comparing patients with active cancer versus past cancer who underwent TAVR.


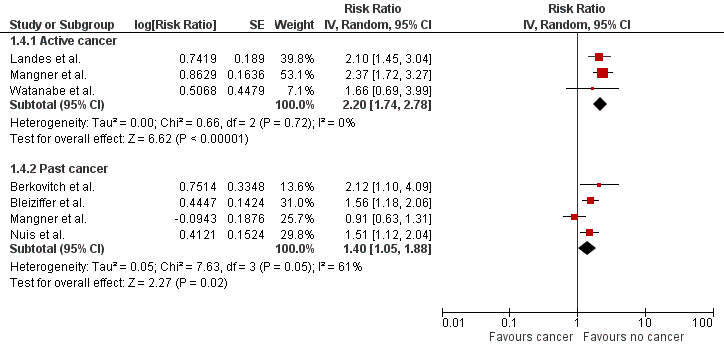


Legend: The pooled risk ratio with 95% confidence intervals were calculated using a random effects model. Weight refers to the contribution of each study to the pooled estimate. Squares and horizontal lines denote the point estimate and 95% confidence interval for each study’s risk ratio. The diamond signifies the pooled risk ratio; the diamond center denotes the point estimate and the width denotes the 95% confidence interval.

**Supplemental Figure 4.** Forest plot for long-term mortality comparing patients with follow-up of 2 years or less versus follow-up of greater than 2 years who underwent TAVR.


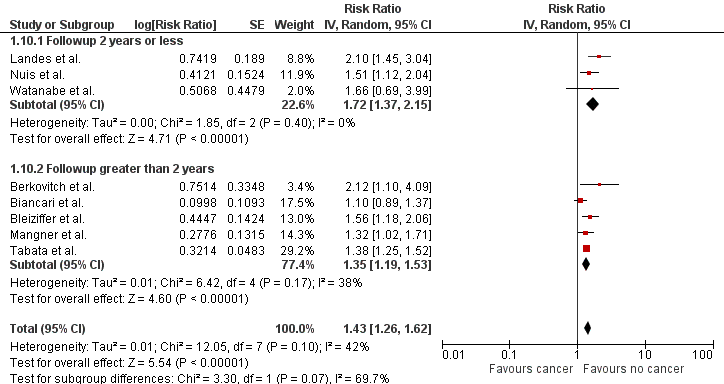


Legend: The pooled risk ratio with 95% confidence intervals were calculated using a random effects model. Weight refers to the contribution of each study to the pooled estimate. Squares and horizontal lines denote the point estimate and 95% confidence interval for each study’s risk ratio. The diamond signifies the pooled risk ratio; the diamond center denotes the point estimate and the width denotes the 95% confidence interval.


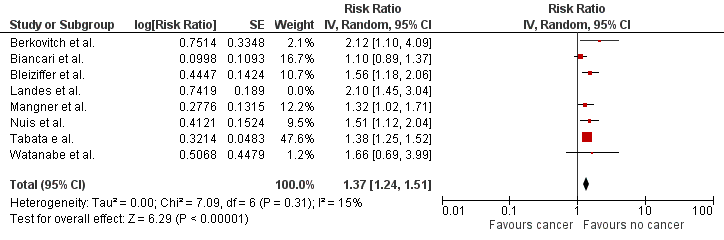
**Supplemental Figure 5.** Leave one out forest plot for long-term mortality comparing patients with and without cancer who underwent TAVR

Legend: The pooled risk ratio with 95% confidence intervals were calculated using a random effects model. Weight refers to the contribution of each study to the pooled estimate. Squares and horizontal lines denote the point estimate and 95% confidence interval for each study’s risk ratio. The diamond signifies the pooled risk ratio; the diamond center denotes the point estimate and the width denotes the 95% confidence interval.


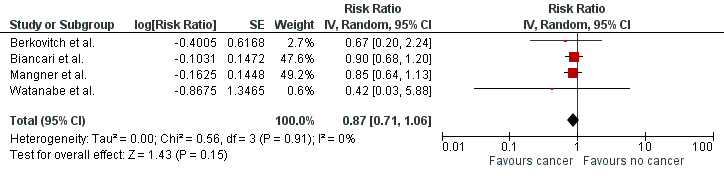
**Supplemental Figure 6.** Forest plot for long-term cardiac mortality comparing patients with and without cancer who underwent TAVR.

Legend: The pooled risk ratio with 95% confidence intervals were calculated using a random effects model. Weight refers to the contribution of each study to the pooled estimate. Squares and horizontal lines denote the point estimate and 95% confidence interval for each study’s risk ratio. The diamond signifies the pooled risk ratio; the diamond center denotes the point estimate and the width denotes the 95% confidence interval.

**Supplemental Figure 7.** Forest plot for long-term secondary endpoints comparing patients with and without cancer who underwent TAVR


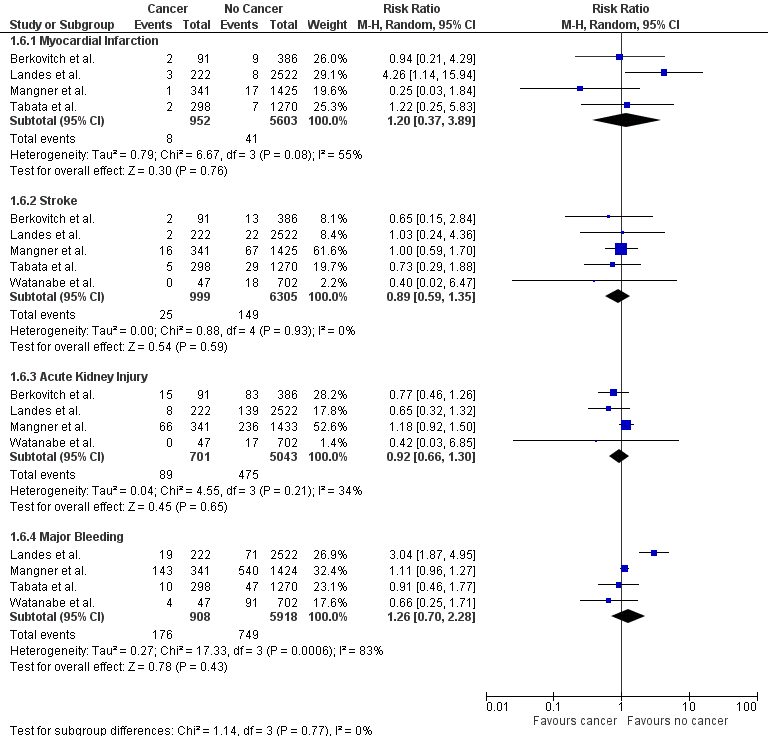


Legend: The pooled risk ratio with 95% confidence intervals were calculated using a random effects model. Weight refers to the contribution of each study to the pooled estimate. Squares and horizontal lines denote the point estimate and 95% confidence interval for each study’s risk ratio. The diamond signifies the pooled risk ratio; the diamond center denotes the point estimate and the width denotes the 95% confidence interval.

**Supplemental Figure 8.** Funnel plot to assess for publication bias in the studies reporting long term mortality


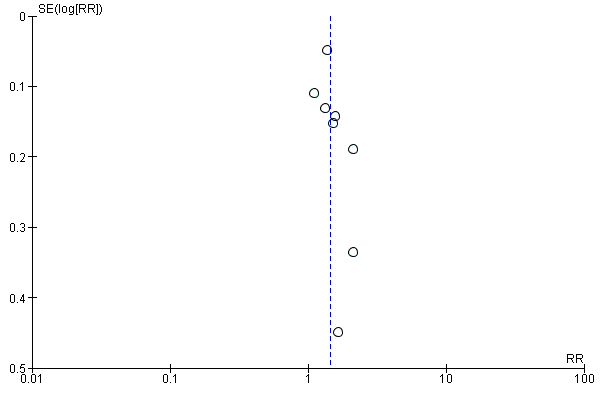


**Supplemental Tables**

**Supplemental Table 1.** Risk of bias in the included studies.

| Modified Newcastle Ottawa Scale | Studies | | | | | | | |
| --- | --- | --- | --- | --- | --- | --- | --- | --- |
|  | Mangner et al, 2018 | Watanabe et al, 2016 | Landes et al, 2019 | Berkovitch et al, 2018 | Biancari et al, 2020 | Bleiziffer et al, 2017 | Nuis et al, 2013 | Tabata et al, 2020 |
| Selection | 4 | 4 | 4 | 4 | 4 | 4 | 4 | 4 |
| Comparability | 1 | 1 | 1 | 1 | 2 | 2 | 2 | 2 |
| Adjustment | Unadjusted | Unadjusted | Unadjusted | Unadjusted | Adjusted | Adjusted | Adjusted | Adjusted |
| Outcome | 3 | 2 | 2 | 3 | 3 | 3 | 2 | 3 |
| **Total (maximum score = 9)** | 8 | 7 | 7 | 8 | 9 | 9 | 8 | 9 |

Legend: for selection, the highest score was 4 based on the representativeness of the exposed cohort, selection of the non-exposed cohort, ascertainment of the exposure, and outcome of interest at the start of the study; for comparability, the highest score was 2 based on comparability of the cohort; and for outcome, the highest score was 3 based on assessment of the outcome, follow-up period and adequacy of the follow-up.

| Author, Year | | Group | LVEF | Gender | | | Age | | | |
| --- | --- | --- | --- | --- | --- | --- | --- | --- | --- | --- |
|  |  | |  | Male | Female | Both | Mean | SD | Median | Range |
| Mangner, 2018 | Cancer | | 57 | 59 | 40 | 99 | 81 | N/A | N/A | 77-84 |
|  | No cancer | | 58 | 628 | 843 | 1471 | 81 | N/A | N/A | 77-84 |
| Watanabe, 2016 | Cancer | | 65.9 | 21 | 26 | 47 | 83 | N/A | N/A | 80-87 |
|  | No cancer | | 65.0 | 232 | 470 | 702 | 85 | N/A | N/A | 82-88 |
| Landes, 2019 | Cancer | | N/A | 138 | 84 | 222 | 78.8 | 7.5 | N/A | N/A |
|  | No cancer | | N/A | 1135 | 1387 | 2522 | 81.3 | 7.1 | N/A | N/A |
| Berkovitch, 2018 | Cancer | | N/A | 47 | 44 | 91 | 79.4 | 8.6 | N/A | N/A |
|  | No cancer | | N/A | 201 | 185 | 386 | 81.8 | 7.0 | N/A | N/A |
| Biancari, 2020 | Cancer | | N/A | 204 | 213 | 417 | 80.6 | 6.6 | N/A | N/A |
|  | No cancer | | N/A | 754 | 959 | 1713 | 81.4 | 6.6 | N/A | N/A |
| Bleiziffer, 2017 | Cancer | | N/A | N/A | N/A | N/A | N/A | N/A | N/A | N/A |
|  | No cancer | | N/A | N/A | N/A | N/A | N/A | N/A | N/A | N/A |
| Nuis, 2013 | Cancer | | N/A | N/A | N/A | N/A | N/A | N/A | N/A | N/A |
|  | No cancer | | N/A | N/A | N/A | N/A | N/A | N/A | N/A | N/A |
| Tabata, 2020 | Cancer | | 53.9 | 148 | 105 | 253 | 80.8 | 5.7 | N/A | N/A |
|  | No cancer | | 54.2 | 594 | 676 | 1270 | 81.1 | 6.7 | N/A | N/A |

**Supplemental Table 2.** Age and gender of patients included.

Legend: LVEF: Left Ventricular Ejection Fraction, SD: standard deviation, N/A: not available

**Supplemental Table 3.** Outcome data from the included studies.

| Author, Year | Outcomes | No cancer | Cancer | Active cancer | Past cancer | P Value |
| --- | --- | --- | --- | --- | --- | --- |
| Mangner, 2018 | Short-term mortality | 1471(112) | 350(17) | 99(6) | 251(11) | 0.18 |
|  | Long-term mortality | 1471(194) | 350(61) | 99(31) | 251(30) | N/A |
|  | Cardiac mortality | 1471(253) | 350(51) | 99(20) | 251(31) | 0.07 |
|  | Non-cardiac mortality | 1471(53) | 350(27) | 99(17) | 251(10) | <0.01 |
|  | Myocardial infarction | 1425 (17) | 341(1) | 96(1) | 245(0) | 0.23 |
|  | Stroke | 1425(67) | 341(16) | 96(4) | 245(12) | 0.96 |
|  | Acute kidney Injury | 1433(236) | 341(66) | 96(20) | 246(46) | 0.41 |
|  | Major Bleeding | 1424(540) | 341(143) | 96(44) | 245(99) | 0.26 |
| Watanabe, 2016 | Short-term mortality | N/A | N/A | N/A | N/A | N/A |
|  | Long-term mortality | 702(45) | 47(5) | 47(5) | N/A | N/A |
|  | Cardiac mortality | 702(5) | 47(0) | 47(0) | N/A | N/A |
|  | Non-cardiac mortality | 702(40) | 47(3) | 47(3) | N/A | N/A |
|  | Myocardial infarction | N/A | N/A | N/A | N/A | N/A |
|  | Stroke | 702(18) | 47(0) | 47(0) | N/A | 0.24 |
|  | Acute kidney Injury | 702(17) | 47(0) | 47(0) | N/A | 0.28 |
|  | Major bleeding | 702(91) | 47(4) | 47(4) | N/A | 0.38 |
| Landes, 2019 | Short-term mortality | 2522(80) | 222(4) | 222(4) | N/A | 0.47 |
|  | Long-term mortality | 2522(157) | 222(29) | 222(29) | N/A | N/A |
|  | Cardiac mortality | N/A | 222(9) | 222(9) | N/A | N/A |
|  | Non-cardiac mortality | N/A | 222(24) | 222(24) | N/A | N/A |
|  | Myocardial infarction | 2522(8) | 222(3) | 222(3) | N/A | 0.36 |
|  | Stroke | 2522(22) | 222(2) | 222(2) | N/A | 1 |
|  | Acute kidney injury | 2522(139) | 222(8) | 222(8) | N/A | 0.28 |
|  | Major bleeding | 2522(71) | 222(19) | 222(19) | N/A | <0.01 |
| Berkovitch, 2018 | Short-term mortality | 386(20) | 91(1) | N/A | 91(1) | 0.15 |
|  | Long-term mortality | 386(24) | 91(12) | N/A | 91(12) | N/A |
|  | Cardiac mortality | 386(19) | 91(3) | N/A | 91(3) | N/A |
|  | Non-cardiac mortality | 386(25) | 91(10) | N/A | 91(10) | N/A |
|  | Myocardial infarction | 386(9) | 91(2) | N/A | 91(2) | 0.97 |
|  | Stroke | 386(13) | 91(2) | N/A | 91(2) | 0.85 |
|  | Acute kidney injury | 386(83) | 91(15) | N/A | 91(15) | 0.39 |
|  | Major bleeding | N/A | N/A | N/A | N/A | N/A |
| Biancari, 2020 | Short-term mortality | 1713(50) | 417(13) | N/A | N/A | 0.78 |
|  | Long-term mortality | 1713(1016) | 417(271) | N/A | N/A | N/A |
|  | Cardiac mortality | 1713(719) | 417(188) | N/A | N/A | N/A |
| Tabata, 2020 | Long-term mortality | 1232(618) | 292(202) | 43(36) | 249(164) | N/A |
|  | Myocardial infarction | 1270(7) | 298(2) | N/A | N/A | N/A |
|  | Major bleeding | 1270(47) | 298(10) | N/A | N/A | N/A |
|  | Stroke | 1270(29) | 298(5) | N/A | N/A | N/A |
| Bleiziffer, 2017 | Long-term mortality | Risk ratio 1.56, 95% confidence interval 1.18-2.05, p=0.002 | | | | |
| Nuis, 2013 | Long-term mortality | Risk ratio 1.51, 95% confidence interval 1.12-2.04. p=0.007 | | | | |

Legend: N/A: Not Available
